# Supplementary material for: Binge Eating and Obesity Differentially Alter the Mesolimbic Endocannabinoid System in Rats
Source: Int J Mol Sci. 2025 Jan 31;26(3):1240. doi: 10.3390/ijms26031240 (PMC11818181; doi:10.3390/ijms26031240)
Supplement: Supplementary file 1 [file ijms-26-01240-s001.zip › ijms-3411845-supplementary.docx]

**Supplementary Figure and legend – Schoukroun et al.**

**Figure S1:** Free-choice high-fat high-sucrose as a model of obesity and binge eating

This figure shows data for cohort B. **(A)** The intermittent access (IA) to palatable food induced a higher consumption of fat and sucrose during the 2h access throughout the entire duration of the protocol, in comparison with the continuous access (CA) group. **(B)** Rats in the IA group consumed significantly more sucrose and fat during the first hour (1^st^) of access versus the second hour (2^nd^). In contrast, no significant differences were observed for the CA group. **(C)** Weight gain throughout the diet was similar between groups, and the CA group showed an increased adiposity. NA : No-access (n = 10), CA : Continuous access (n = 10), IA : Intermittent Access (n = 10). Results are expressed as mean ± SEM. * p < 0.05 ; ** p < 0.01 ; *** p < 0.001.
